# Automatic pathway building in biological association networks

**DOI:** 10.1186/1471-2105-7-171

**Published:** 2006-03-24

**Authors:** Anton Yuryev, Zufar Mulyukov, Ekaterina Kotelnikova, Sergei Maslov, Sergei Egorov, Alexander Nikitin, Nikolai Daraselia, Ilya Mazo

**Affiliations:** 1 Ariadne Genomics Inc, 9700 Great Seneca Hwy, Suite 113, Rockville, MD 20850, USA

## Abstract

**Background:**

Scientific literature is a source of the most reliable and comprehensive knowledge about molecular interaction networks. Formalization of this knowledge is necessary for computational analysis and is achieved by automatic fact extraction using various text-mining algorithms. Most of these techniques suffer from high false positive rates and redundancy of the extracted information. The extracted facts form a large network with no pathways defined.

**Results:**

We describe the methodology for automatic curation of Biological Association Networks (BANs) derived by a natural language processing technology called Medscan. The curated data is used for automatic pathway reconstruction. The algorithm for the reconstruction of signaling pathways is also described and validated by comparison with manually curated pathways and tissue-specific gene expression profiles.

**Conclusion:**

Biological Association Networks extracted by MedScan technology contain sufficient information for constructing thousands of mammalian signaling pathways for multiple tissues. The automatically curated MedScan data is adequate for automatic generation of good quality signaling networks. The automatically generated Regulome pathways and manually curated pathways used for their validation are available free in the ResNetCore database from Ariadne Genomics, Inc. [1]. The pathways can be viewed and analyzed through the use of a free demo version of PathwayStudio software. The Medscan technology is also available for evaluation using the free demo version of PathwayStudio software.

## Background

The advances of the high-throughput technologies and enormous growth in number of experimentally determined interactions have necessitated the development of a database storing molecular interactions network. Such a database can be used to develop an algorithm that interprets the high-throughput data, for analysis of properties of biological networks, and for automatic prediction of biological pathways. Yet the most reliable knowledge about molecular interactions and pathways currently exists in the form of peer-reviewed scientific literature written in the form of human language scientific jargon. The exponential growth of such literature in the last 20 years has made manual fact extraction nearly impossible as well as highly expensive. To address this problem, several text-mining algorithms for automatic fact extraction have been developed. See the introduction in reference [2] for a thorough review of text-mining methods.

In brief, the text-mining algorithms for relations extraction can be classified into those that use simple statistical co-occurrence [3,4], pattern matching [5,6], or full-sentence parsing algorithms [2,7]. Every text-mining algorithm can be characterized by: a) recovery rate, which measures how many facts it recovers compared to the human curator from the same number of sentences, and b) accuracy rate measuring the percentage of false positives among recovered facts. The co-occurrence algorithms recovery rate depends entirely on the quality of term recognition that is the dictionaries and can be as high as 100%; however, their accuracy rate does not rise above 50%. On the contrary, the recovery rate of the full-sentence parsing methods usually does not rise above 50%, yet they have much better accuracy rates. The pattern matching algorithms tend to have intermediate performance between co-occurrence and full-sentence parsing.

The text-to-knowledge technology called Medscan is a natural language processing full-sentence parsing system developed by Ariadne Genomics [8,9]. Medscan can derive the relations between objects only within a scope of one sentence. It recognizes the complete syntactic structure of an English language sentence in order to determine the relation between entities. The core of the technology is domain independent and is capable of finding relations between any types of objects derived purely from the semantic and lexical structure of a sentence. However, Medscan has been tuned and curated a great deal towards the language of scientific papers in the field of Molecular Biology. The performance of Medscan has been reported previously [9]. Medscan recovers about 60% of all relations per sentence in the text. This rate enables nearly 100% detection for all facts that are repeated more than once in the literature corpus. Most single-reference facts are usually re-stated several times in a single full-text article, enabling Medscan to recover unique single-referenced relations with high certainty as well. We estimate that the most recent Medscan pipeline, version 1.8, extracts 90% of all facts described in the scientific literature. About 10% of all relations found by Medscan are false positive. This false positive rate has been thoroughly measured in [9] and is used by this work as the threshold for eliminating false positives in most automatic curation rules.

Most information extraction systems in the biomedical domain suffer from the redundancy of extracted relations and a false positive rate that interferes with further network analysis of the extracted data. An aggregation of automatically extracted relations has been proposed as a solution to overcome some of these problems [10]. In the first part of the paper, we describe several methods for automatic relation consolidation and curation in the ResNet database. ResNet is the database of Biological Association Networks (BANs) available for purchase from Ariadne Genomics. It contains molecular interaction data extracted by Medscan technology, as well as the interaction data available from the public sources such as Entrez Gene, BIND, and HPRD. The ResNet database schema is generic and capable of storing, retrieving, and navigating any type of heterogeneous networks. BANs in ResNet contain annotation for mammalian proteins, small chemicals, and functional classes as graph vertices linked with different types of relations as graph edges. The edges also contain annotations about relation types and references to a literature source where the relation was extracted by Medscan.

Currently, Medscan technology extracts relations between proteins, small molecules, protein functional classes, cell processes, and diseases. These relations can be divided into two major classes: direct physical interactions and indirect regulation events. The direct physical interactions include three types of relations:

• Binding (*Binding*)

• Protein modification (*ProtModification*)

• Promoter binding (*PromoterBinding*).

The indirect interactions include:

• Regulation (*Regulation*)

• Expression regulation (*Expression*)

• Molecular transport regulation (*MolTransport*)

• Molecular synthesis regulation (*MolSynthesis*).

*Regulation *is the most abundant relation type in ResNet, reflecting the most common way scientists express their thoughts about protein signaling. The ResNet database contains information about proteins from human, mouse and rat organisms. The orthologs from these three species are merged in one node in ResNet (10). Relations found by Medscan are annotated by organism, but algorithms described in this paper do not use this information. Complete statistics for every relation type in ResNet database are presented in Table 1.

Medscan also extracts information about the relation direction, effect on a target molecule, and mechanism of action if this information is present in a sentence describing the relation. This additional information is recorded in attributes "Effect" and "Mechanism" for the extracted relation. The attribute "Mechanism" is required for *ProtModification *relation and is derived from the verbs used in the sentence, such as "phosphorylate," "glycosylate," "dephosphorylate," etc. The "Effect" attribute has the following values: "positive," "negative," and "unknown." Every relation is recorded as an individual XML object in the output of the Medscan. During an import into the ResNet database, two relations are considered the same and merged if they connect the same pair of nodes in the same direction and have both the same effect sign and the same mechanism. If none of these conditions are met, the relation is not merged and is recorded as a new relation.

Relations in ResNet are generated from multiple literature sources including the entire PubMed database containing 13,000,000 scientific abstracts and 43 publicly available full-text journals. Medscan processes individual sentences and does not accumulate the information about all other relations in the literature corpus during parsing. As a consequence, the same biological relation expressed differently by different authors will be recorded as two different relations by Medscan. Also, many sentences contain only partial descriptions of the relations from the Medscan output. Historically, the relations between proteins are usually first detected as regulation events and then as an exact mechanism of action through binding, protein modification, promoter binding; otherwise, a more precise mechanism for indirect regulation is established in later publications. All these reasons create many true but redundant relations in the ResNet database after the import of PubMed data processed by Medscan. Medscan's 10% false positive rate further complicates the picture, especially for highly-cited relations. Most Medscan false positives are due to an incorrectly recorded effect or direction for a relation. The most dramatic example of the heterogeneity produced by recording of the natural language sentences into a set of formalized relations can be found for interactions between p53 and MDM2 proteins. Medscan finds 2,894 sentences describing a relation between these two proteins in the entire literature corpus. The information from these sentences is interpreted by Medscan as 29 different relation types, including nine false positive relations (data not shown).

To facilitate the creation of new algorithms for analysis of ResNet data and for interpretation of the experimental data using ResNet, we have to reduce the complexity of Biological Association Networks produced by Medscan. Ideally, every linked protein pair should contain only a single link in one direction. The first part of this paper describes algorithms for automatic curation of the Medscan data converting BANs into a simple graph. The procedure also reduces the number of false positive links in ResNet. We used the data obtained after the curation procedure to automatically build pathways containing links from the purified BAN. We show that the automatically curated ResNet data contains a sufficient amount of information to build thousands of signaling pathways. The algorithm for pathway building is also described.

## Results

### ResNet curating algorithms

The complete set of rules and results for automatic curation are described in Additional file 1 and Table 2. These rules were developed by biology experts after extensive inspection of ResNet data produced by the Medscan. The accuracy of curation of every rule was also manually evaluated (refer to Additional file 1 legend). Most cleaning rules were designed to remove Medscan errors. Yet some cleaning rules aim to overcome the jargon of the molecular biologist. For example, it is common practice to write that insulin phosphorylates some intracellular protein. The phrase always means that insulin induces the protein phosphorylation indirectly, but semantically it implies direct phosphorylation by insulin. Other examples include the sentences describing interaction between cytokines. Almost exclusively they mean the functional interaction of their downstream signaling pathways, but semantically they imply physical binding.

All merging rules were designed to overcome the shortcomings due to the historical nature of scientific literature and the differences in the way scientists describe the interaction between proteins. For example, it is common to simply mention that p53 regulates MDM2 without specifying that it actually positively regulates the MDM2 expression by binding to the MDM2 promoter. Yet other sentences describe p53 binding to the MDM2 promoter and still others describe p53 regulation of the MDM2 expression. Medscan is "unaware" of other facts during text processing, and compilation of all these different facts can be performed only by analyzing the entire collection of available ResNet facts.

Several curation rules generate new relation types in ResNet. For example, the *DirectRegulation *type symbolizes that one protein binds and regulates another. *DirectRegulation *can have an attribute mechanism that further specifies the regulation mechanism through binding or protein modification type. To avoid propagating false positive relations, we allowed merging only if the target relation had a number of references above the Medscan false positive rate as compared to the relation targeted for deletion (Additional file 1). Doing this ensured that the target control was always a true positive. An exception was made for merging into the *PromoterBinding *relation. We found that the cleaning rule for *PromoterBinding *relation, which allowed having only transcription factors as regulators, almost completely eliminated false positives for this relation. Therefore, the merge of the *Expression *relation with the *PromoterBinding *relation was always allowed.

### Building Ligand-Receptor regulomes

The pathway building algorithm uses the core sub-network building procedure described in the Materials and Methods section. Ligand regulomes were defined as a set of proteins regulated by either ligand or its receptor. First, we found all possible pairs of ligands physically interacting with receptors in the curated ResNet data. There were 368 such interactions in ResNet. Downstream proteins were selected as proteins linked to either ligand or receptor by any of the indirect regulatory links or as proteins physically interacting with a receptor. To ensure a high confidence of regulome pathways, we used only physical interactions that had more than five references. If pathway building with only high-confidence relations was impossible, the cutoff reference count was gradually relaxed below five until the pathway construction became possible. This approach yielded 351 regulomes for 146 ligands and 139 receptors from the entirety of ResNet. Among them 106 pathways were constructed only from relations with more than five references.

We have monitored how the average number of nodes in pathways was changing with a reference cut-off and found that it did not change much while the cut-off was increased. For example, the average number of nodes in a pathway was 74 nodes per pathway with no cutoff, while pathways had on average 52 nodes with a reference cutoff equal to 10. The average number of relations, however, changed dramatically from 465 in pathways with no cutoff to 192 in pathways with a cutoff equal to 10. The overall number of generated regulomes dropped from 350 pathways generated with no cutoff to 57 generated only with relations that had more than 10 references. We interpret these results in the following way: most pathways have backbone interactions that are studied equally well; i.e., they have about the same number of references. Once the reference cutoff is increased above the average citation index of these backbone interactions, the pathway cannot be built. In addition to backbone interactions, every pathway has a large number of less-studied relations. These relations mostly connect the same nodes that form a well-cited backbone. Such less-cited interactions are lost more rapidly with an increase of reference cutoff, but their loss does not affect pathway integrity.

### Validation of predicted regulomes pathways

To validate automatically generated regulome pathways, we have compared them with the set of 144 pathways manually constructed, based on review articles. One hundred-six pairs of regulome pathways and manually curated pathways were identified as having the same ligand-receptor pair and were thus valid for comparison. We found that, on average, pathways constructed for the same ligand have 62.1% in node overlap and 18.2% in relation overlap. We also calculated the *p*-value of the overlap between the pair of regulomes and the manually built pathway using the Fisher exact test. The *p*-value shows the probability of finding the particular regulome pathway among all pathways in the database, as compared to random sampling. All *p*-values for all tested pathway pairs were smaller than 0.0001, indicating that the similarity between two pathways is not due to random chance. The distribution of the node overlap among test pathways pairs is shown in Figure 2. The example of an automatically built pathway for IL1 and its receptor is shown on Figure 4.

To further validate predicted pathways we have assessed that pathway construction was possible using proteins co-expressed only in one tissue. The publicly available gene expression dataset for 79 tissues was used to select tissue-specific proteins. The expression threshold to select proteins expressed in a tissue was intentionally stringent. The approach described in the Materials and Methods section has yielded 7,585 pathways for 79 tissues, 122 ligands, 103 receptors and 217 ligand-receptor pairs. The distribution of number of pathways built for every tissue is shown in Figure 5. The comparison of the protein composition among 7,585 pathways revealed that 5,692 pathways had unique protein compositions.

To obtain additional support for the biological relevance of the tissue-specific pathways, we have compared the number of pathways built for ligands specific to the central nervous system (CNS) in different brain tissues to the number of pathways built for the same ligands in the immunological tissues. We found that, for eight CNS-specific ligands, the algorithm built 143 pathways in 23 CNS-related tissues, while only 93 pathways were built for the same ligands in 21 immune system tissues. Thus, there were 1.5 times more pathways per tissue built in CNS, compared to the immune system. Similarly, for 36 immunological ligands, 650 pathways were built in immune system tissues and only 484 pathways in CNS tissues. Thus, there were 1.34 times more pathways per tissue built in the immune system, compared to CNS for immunological ligands (Figure 6).

## Discussion

We describe the computational approach to automatically build signaling pathways using the network database of Biological Associations extracted from scientific literature. The approach is based on a simple notion that the propagation of a regulatory signal is mediated by means of physical interactions in a living cell. The automatic curation step is required for the pathway reconstruction in order to consolidate extracted relations in the database. Therefore, this paper also describes rules for automatic curation. Without curation the pathway prediction algorithm incorporates false positive relations, making pathways bigger on average (data not shown). Most importantly, however, without relation consolidation performed by merging rules during automatic curation, the pathway reconstruction algorithm becomes impractical. Many proteins are connected by multiple relations in the dataset produced by Medscan, as explained in the Introduction. Without their merging pathway reconstruction, algorithms would have to "choose" what relation to include into every pathway. This process would slow down the algorithm significantly.

The automatic curation algorithms produce better quality networks suitable for analysis by other algorithms developed for interpretation of the experimental data. As much as the Medscan natural processing technology can be viewed as an automation of the reading process, the automatic curation can be viewed as automation of the literature reviewing process that uses the domain-specific knowledge for better interpretation of the facts recorded in natural language.

Most of our automatic curation rules can be generalized for curation of any kind of BANs and not only the networks produced by Medscan technology. However, we believe that the reference count thresholds reported in Additional file 1 of this paper are specific to Medscan technology. The thresholds values for other BANs will most certainly depend on the actual accuracy of information in the curated database. We developed automatic curation from the need to overcome problems caused by scientific jargon and because of the historical nature of the scientific literature. The historical problems are likely to appear for any BAN derived from the scientific literature, including manually curated databases. Any database that monitors scientific literature for a sufficiently long period should accumulate redundancy due to historical developments and curation errors during the database life cycle. To refresh knowledge accumulated in the aged database or to merge the older knowledge into the new database, curation of the old database is necessary.

The algorithms for automated pathway building were developed from the need to present BAN as a set of sub-networks. Such sub-networks can be used for analysis of the experimental data from gene expression microarray and other high-throughput methods. The ultimate goal for the analysis of experimental data is to find the sub-network(s) most affected in the experiment. This process can be done by directly traversing the entire network while looking for the most active sub-networks [11]. This approach, however, finds sub-networks without considering biological functionality, and thus their functional interpretation has to be completed separately. The alternative approach is to pre-cut BAN into multiple functional blocks and to find the most active sub-networks among them.

The core sub-network building procedure presented in this paper was used for successful generation of ligand regulomes. Yet it can also be used for construction of other types of pathways. For example, we have used the Gene Ontology biological process annotation to build pathways describing biological processes (data not shown). Another possibility is to use the list of proteins related to a disease and build disease-association pathways. The use of the trimming procedure described in the Materials and Methods section as the second step of the pathway reconstruction algorithm is optional and can be skipped for non-signaling pathways. Also, the sub-network may be allowed to include indirect regulations if no sufficient number of physical interactions exists for a given protein list.

The biological functionality of the result pathway is solely determined by the input protein list. The biological relevance of regulome pathways is achieved in part by using the high quality protein classification but also heavily depends on the input list. Our approach separates the construction of a protein list from pathway building and automates the latter step. This approach allows for focusing manual curation efforts on the development of functional protein lists using non-network information such as sequence homology, disease and phenotype association, or protein clusters in other types of networks.

## Conclusion

We demonstrated that the data extracted automatically by MedScan technology can be further automatically curated to generate a high-quality molecular interaction dataset. The quality of the automatically curated ResNet is sufficient to automatically reconstruct thousands of biologically relevant signaling pathways for multiple mammalian tissues using basic principles known for signaling pathways *in-vivo*.

## Methods

### Protein classification in ResNet

We have used Gene Ontology [12] and Entrez Gene annotation (NCBI) to classify all proteins in the ResNet database in 26 groups. The classes were designed based on the needs for ResNet curation and for pathway building. The statistics of our protein classification and the correspondence of our classes to Gene Ontology groups are described in Additional file 2.

The group assignment was done automatically by parsing Entrez Gene annotation and traversing the Gene Ontology tree so as to include child classes, if necessary. The proteins that were not classified by this method were additionally classified by sequence similarity to proteins that have been already classified. We considered two proteins to be paralogs if their amino acid sequence similarity was higher than 30%. The procedure to find protein paralogs has been described previously [13]. Automatic classification was followed by extensive manual curation to resolve conflicting annotation.

The following conflict rules were used for manual curation:

1) Transcription factors cannot be kinases, phosphatases, secreted proteins, ligands, or extracellular matrix proteins;

2) Kinases cannot be phosphatases, transcription factors, or ligands;

3) Ligands cannot be transcription factors, phosphatases, kinases, or nuclear receptors;

4) Phosphatases cannot be kinases, transcription factors, or ligands;

5) Receptors cannot be GPCR, nuclear receptors, transcription factors, or secreted proteins;

6) Nuclear receptors cannot be any other class;

7) GPCR cannot be any other class;

8) Extracellular matrix proteins cannot be secreted proteins, kinases, phosphatases, or ligands.

### Implementation of ResNet curating algorithms

Automatic curation of ResNet database consists of the following procedures:

• Relation conversion

• Relation merging

• False positive elimination for most cited proteins; coherent loop conversion.

The relation conversion changes the relation type or deletes relations between a regulator and a target, according to the conversion rules described in Additional file 1. For example, if a relation is extracted as *ProtModification *by Medscan, but a regulator in this relation is not a kinase, then the relation is converted to *Regulation*. The new *Regulation *relation is also annotated by the property mechanism with the value "Phosphorylation". However, if the original *ProtModification *relation has fewer than five references, the relation is considered a false positive and simply is deleted by the curation program.

The relation merging compresses all relations of one type and direction into a single relation of the same type. Mechanism and Effect properties are transferred from the relation with the largest number of references. The next step merges relations of different types according to merging rules in Additional file 1. For example, if there are *Binding *and *ProtModification *relations between a pair of nodes, then these relations are merged into the *ProtModification *relation. To avoid merging with false positives, in this example, the merge only takes place if the number of *ProtModification *references is at least 1/10 of the number of *Binding *references.

False positive elimination removes one of two directed relations that connect the same pair of nodes in opposite directions. The relation with fewer references is removed only if its reference count is lower than the Medscan false positive rate (10%) of the reference count for another relation. Both nodes must have high connectivity (more than 50%). This rule was introduced in order to suppress the high number of false positives between frequently cited proteins connected by relations with a large number of references. If the relation is described by many sentences in the literature corpus, Medscan is likely to misinterpret one of the sentences and assign the wrong direction to the link.

**Figure 1:**
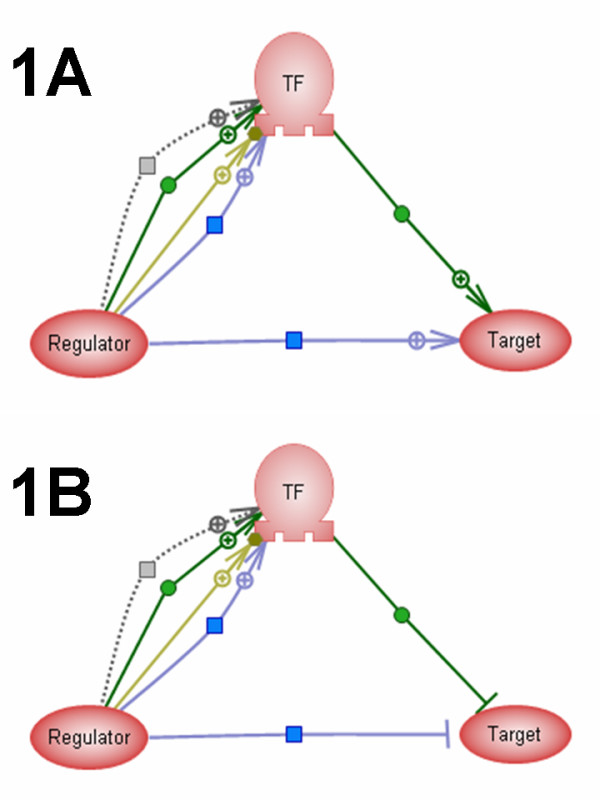
**Examples of the coherent loops used for automatic ResNet curation. **Node TF represents a transcription factor. Green arrows – *PromoterBinding *relations, blue arrow – *Expression *relation, grey arrow – *Regulation*, Brown arrow – *MolSynthesis*. The coherent loop conversion rule converts the expression link from Regulator node to Target node into *Regulation *link. Arrow with "+" indicate positive regulation and negative regulation is shown as "---|". **A**. Coherent loop with all relation having positive effect. Regulator->TF: positive; TF->Target: positive; Regulator->Target: positive; **B. **Coherent loop with negative effects. Regulator->TF: positive; TF->Target: negative. Regulator->Target: negative. Other two types of coherent loops have following configurations: Regulator->TF: negative; TF->Target: positive; Regulator->Target: negative. Regulator->TF: negative; TF->Target: negative; Regulator->Target: positive.

**Figure 2:**
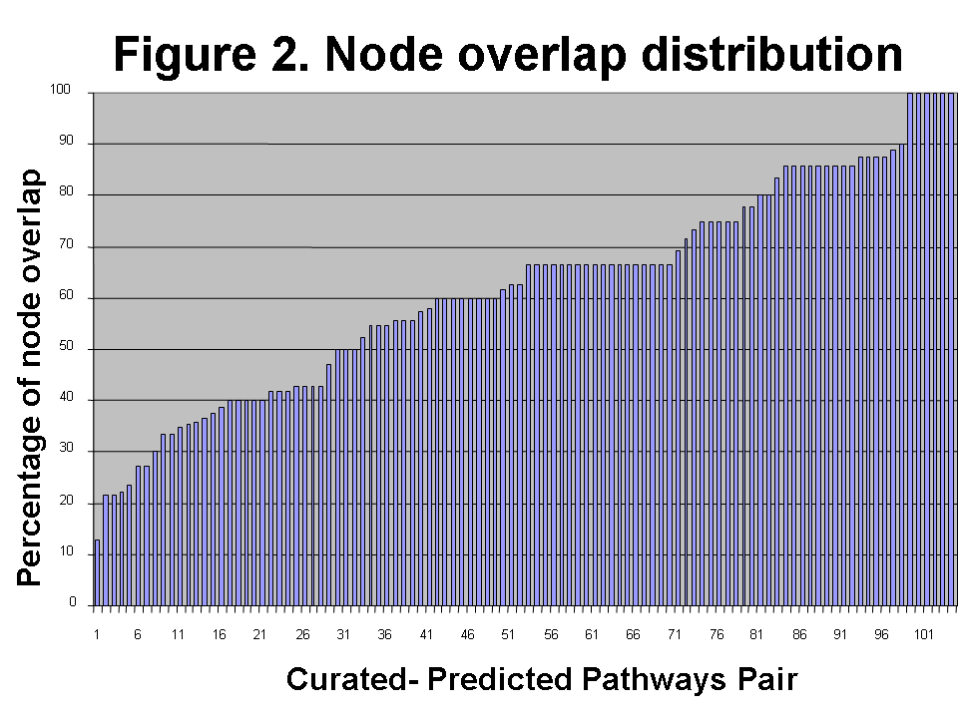
**Distribution of node overlaps between manually curated pathway and equivalent regulome pathway with the same ligand-receptor pair. **X axis – every point represents a pair of manually curated and automatically built pathways, Y axis – number of nodes in common between two pathways.

**Figure 4:**
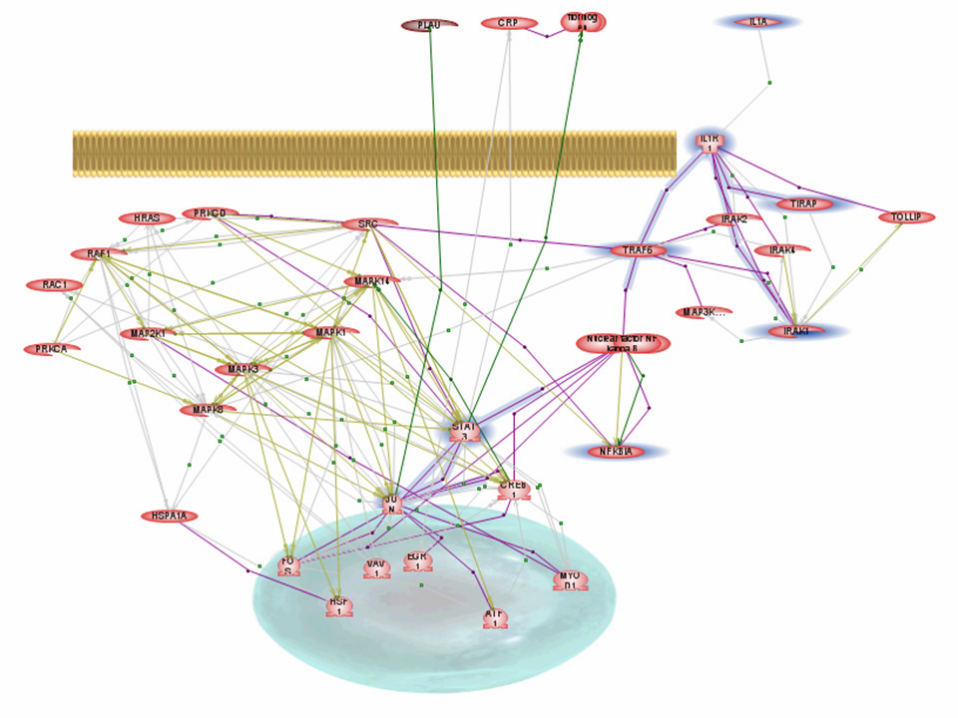
**Automatically built pathway**. Nodes and links in common with manually curated IL-1 pathway shown on Figure 3 are highlighted in blue. Note that the set of proteins unique to automatically built pathway represents a classical MAP kinase cascade. It has been suggested only recently that the IL-1 receptor appears to activate a MAP kinase cascade by interaction with other members of the Toll-like receptor superfamily [17]. Obviously, older review articles used for construction of the manually curated IL-1 pathway did not mention this information. For graph legend see figure 3.

**Figure 5:**
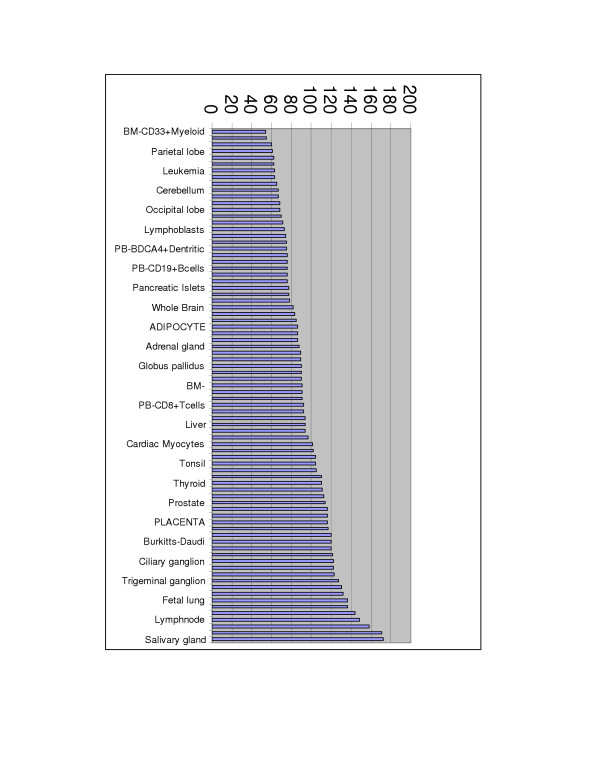
**Distribution of number of Regulome pathways build for 79 tissues based on the tissues gene expression profile. **The tissue-specific pathway construction is described in the Materials and Methods section.

**Figure 6:**
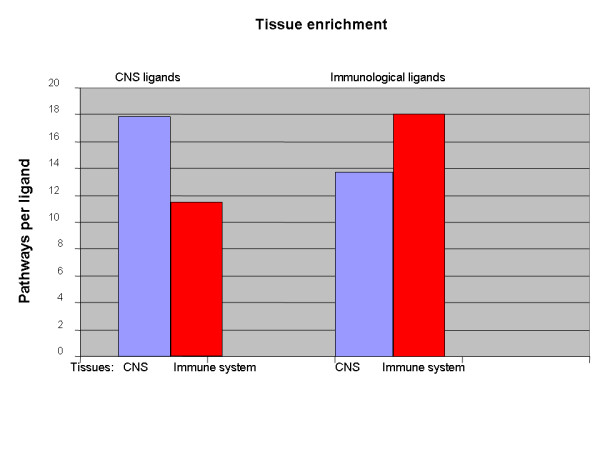
**Increase in the number of pathways predicted for tissue-specific ligands in different tissue types. **The number of pathways is normalized to the number of tissues in every tissue type. Twenty-one immune tissues names listed in [16]: Appendix, BM-CD105+Endothelial, BM-CD33+Myeloid, BM-CD34+, BM-CD71+Early Erythroid, Bone marrow, Burkitts-Daudi lymphoma, Burkitts-Raji lymphoma, Leukemia chronic myelogenous(k562), Leukemia lymphoblastic(molt4), Leukemia promyelocytic(hl60), Lymphnode, Lymphoblasts, PB-BDCA4+Dentritic, PB-CD14+Monocytes, PB-CD19+Bcells, PB-CD4+Tcells, PB-CD56+NKCells, PB-CD8+Tcells, Thymus, Whole blood. Twenty-three CNS tissues names listed in [16]: Amygdala, Caudate nucleus, Cerebellum, Cerebellum peduncles, Ciliary ganglion, Cingulate cortex, DRG, Fetal brain, Globus pallidus, Hypothalamus, Medulla oblongata, Occipital lobe, Olfactory bulb, Parietal lobe, Pituitary, Pons, Prefrontal cortex, Subthalamic nucleus, Superior Cervical Ganglion, Temporal lobe, Thalamus, Trigeminal ganglion, Whole Brain. 8 CNS specific ligands from ResNet database: BDNF (brain derived neurotrophic factor), CNTF (ciliary neurotrophic factor), GDNF (glial cell line derived neurotrophic factor), GPI autocrine motility factor, leptin, NGFB (nerve growth factor beta), NPTX1 (neuronal pentraxin I), NTF3 (neurotrophin 3). List of 36 immunological ligands is not shown.

Examples of coherent loops are shown in Figure 1. The existence of a coherent loop suggests that the *Expression *relation is indirect and mediated by two other relations in the loop. In such loops the *Expression *relation is converted to *Regulation *with the Expression mechanism. The rule was designed to reduce the number of indirect *Expression *links and increase the proportion of PromoterBinding links in the combined Expression regulation network in ResNet. This combined network is used by several algorithms interpreting microarray expression data in Ariadne software. One algorithm finds significant transcriptional regulators for the differentially expressed genes [14]. Another identifies contradictions between the regulatory network and the gene expression data [15].

### Implementation of algorithm for pathway prediction

The complete ResNet dataset was exported from the database after automatic curation in RNEF XML format (ResNet exchange XML format). The database XML dump was converted into a set of keys uniquely describing the relation type and number of references for every relation. A key contains information about two connected nodes, direction of the link, regulatory effect, and mechanism of action. This key conversion allowed fast reading of the ResNet data into the computer's memory. The pathway algorithms were written using C++ STL library from Microsoft in Microsoft Visual Studio 7.0. Two proteins were linked by only one physical link in a pathway in one direction. The algorithm has two steps. First, the program finds all physical interactions between proteins from an input list, thus creating a sub-network from ResNet data. The second step is called the trimming procedure. It removes unlinked nodes and trims the sub-network into a configuration of a signaling pathway. For trimming, all proteins were divided into two groups. The regulator-only group contains ligands, receptors, GPCR, nuclear receptors, and secreted proteins. The proteins from these classes were not allowed to be targets in the sub-network. (Their in-degree, or number of incoming links, must be zero in the sub-network.) The target-only group contains cytoskeletons, transporters, metabolic enzymes, ubiquitin ligases, transcription factors, and ligands. The proteins from these classes were not allowed to be regulators in the sub-network. (Their out-degree, or number of out-going links, must be zero in the sub-network.)

1) The trimming procedure removed all nodes according to following criteria: All nodes with zero in-degree except protein from the regulator-only group. This rule removed all nodes that were not regulated by any other vertices in a pathway;

2) Any proteins that have an out-degree equal to zero except proteins from the target-only group. This rule removed nodes that did not regulate other nodes in the pathway;

3) Any proteins connected to a pathway only with single *Binding *link except proteins from the target-only group. This rule removed nodes that did not regulate other nodes in the pathway;

4) Isolated pairs of linked nodes that were not connected to other proteins in the pathway. This rule converts a pathway into a single connected graph.

Trimming continued for several cycles until no protein could be removed according to the criteria listed above, or until no proteins remained in the pathway.

### Pathway construction by manual curation

One-hundred forty-four pathways were built manually and used as a reference set for comparison with algorithm results. Manual pathways were constructed using relations from the ResNet database and protein lists were compiled from the review articles describing signaling from 144 ligands. Medscan pipeline version 1.7 was used to create ResNet data for manually curated pathways. The missing relations were added manually and supplied with reference information, together with curator names to distinguish them from relations found by Medscan. Four-hundred sixty-four new relations were added and 1,652 were found in ResNet using the Build Pathway tool in PathwayStudio Central™ software from Ariadne Genomics, Inc.

### Construction of tissue-specific pathways

The gene expression data for 79 tissues was taken from a publicly available data set at the NCBI gene expression omnibus [16]. Each sample was normalized by the Fisher Z-transformation, and the expression value for each gene was calculated as an average between two normalized sample values available from the dataset. We have calculated the threshold equal to -0.257 from the assumption that every tissue should contain not more than 80% of all proteins. During the threshold calculation, we found that salivary gland tissue contain the lowest normalized expression values; therefore, only this tissue had 80% of all genes. On average, every tissue had 56% of all genes above the threshold.

The Regulome pathways were constructed for every tissue, as described in the previous section, and every Ligand-Receptor pair was available from ResNet. Every pathway was built from proteins expressed in one tissue. The reference cutoff was determined automatically for every pathway as the maximum number of references allowing the pathway construction. The average reference cutoff for 7,585 tissue-specific pathways was four references. Our algorithm constructed 7,585 tissue-specific pathways in about 25 hours on the 3 Gz Pentium 4 PC.

## Abbreviations

BIND – Biomolecular Interaction Network Database

HPRD – Human Protein Reference Database

BANs – Biological association networks

XML – Extensible Markup Language

CNS – central nervous system

NCBI – National Center for Biotechnology Information

GPCR – G-protein coupled receptor

RNEF XML – ResNet exchange XML

STL – Standard Template Library

## Authors' contributions

AY designed the curation rules, designed and implemented pathway reconstruction algorithm, and wrote the manuscript.

ZM implemented the ResNet curator.

EK built manual pathways and performed comparisons with automatically built pathways.

SM proposed the coherent loop conversion rule.

SE designed and implemented several curation rules.

AN designed and developed the API interface for the ResNet curator.

ND designed several curation rules.

IM was a general manager for the project and proposed several curation rules.

## Supplementary Material

Additional file 1 **Rules and rule results for automatic ResNet curation. **The automatic curation rules are detailed in the Materials and Methods section. Abbreviations: TF – transcription factors; NR – nuclear receptors. The row order represents the order in which the rules were applied during the automatic curation. Rules named after the control type correspond to the merging of relation of the same type connecting two entities in the same direction but having different effect or mechanism. These rules are described in Materials and Methods section. Percentage in the second column was calculated as fraction of the total relations of the type subjected to the curation rule. Combination of all rules deletes or converts about 30% of all relations in the ResNet database. Click here for file

Additional file 2 **Protein classification in ResNet and its correspondence to GO annotation.** Click here for file

## Figures and Tables

**Figure 3:**
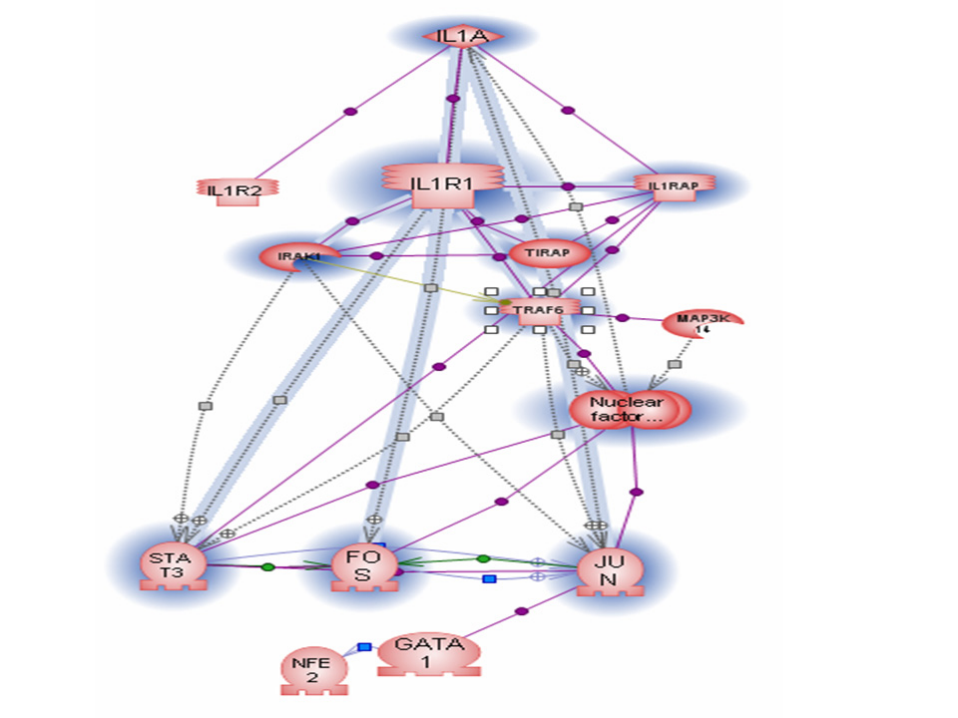
**Manually curated IL-1 pathway.** Nodes and links in common with the automatically built IL-1 regulome pathway shown on Figure 4 are highlighted in blue. Graph legend: IL-1 is shown as red rhombus, Kinases – red sickle shapes, Proteases – brown sickle shapes, receptors – red stick shapes, Transcription factors – red O-shapes, Other proteins – red ovals; Binding – violet links, ProtModification – brown links, DirectRegulation – gray links with green rectangles, PromoterBinding – green links, Expression – blue link.

**Table 1:** Statistics for the ResNet database as of August 21, 2005 generated by Medscan technology version 1.7 before and after automatic curation.

**Name**	**Before autocuration**	**After auto-curation**
Number of proteins with links	11,000	10,845
Number of chemicals with links	37,904	37,904
Number of cell process	897	897
*Binding*	59,149	43,678
*ProtModification*	12,426	11,961
*PromoterBinding*	4,054	3,875
*Regulation*	760,621	509,105
*Expression*	68,711	52,842
*MolTransport*	28,265	20,781
*MolSynthesis*	63,713	55,567
*DirectRegulation*	0	16,772

**Table 2:** Feed-forward and coherent loops statistics. The coherent loops were converted only if the relation between the transcription factor and a target was *PromoterBinding*. Table shows the number of loops with different relation types between the regulator node and the transcription factor node (Figure 1). Coherent loops are feed-forward loops with coherent regulation effects. Because many loops shared the same *Expression *relation between regulator and target, the number of Expression relations converted to regulation is smaller than the total number of coherent loops.

	**Feed-forward loops**		**Coherent loops**	
Relation from Regulator to TF	Number of unique *Expression *relations	Number of loops	Number of unique *Expression *relations	Number of loops

*DirectRegulation*	3242	4849	1109	1345
*Expression*	8911	13468	3638	4600
*MolSynthesis*	231	233	95	96
*PromoterBinding*	591	767	124	133
*Regulation*	10592	17246	4359	5646
**Total**	26689	40588	7411	12023
