# Supplementary material for: Automatic pathway building in biological association networks
Source: BMC Bioinformatics. 2006 Mar 24;7:171. doi: 10.1186/1471-2105-7-171 (PMC1435941; doi:10.1186/1471-2105-7-171)
Supplement: Additional file 1 — Rules and rule results for automatic ResNet curation. The automatic curation rules are detailed in the Materials and Methods section. Abbreviations: TF – transcription factors; NR – nuclear receptors. The row order represents the order in which the rules were applied during the automatic curation. Rules named after the control type correspond to the merging of relation of the same type connecting two entities in the same direction but having different effect or mechanism. These rules are described in Materials and Methods section. Percentage in the second column was calculated as fraction of the total relations of the type subjected to the curation rule. Combination of all rules deletes or converts about 30% of all relations in the ResNet database. [file 1471-2105-7-171-S1.doc]

**Additional file 1. Rules and their results for automatic ResNet curation.** The automatic curation rules are detailed in the Materials and Methods section. The cutoff equal to five in the number of references for certain relations was determined by measuring the dependency of false positive rate from the number of references. We found that the false positive rate drops significantly below 5% among relations with number of reference more than five (data not shown). The cutoff of 10% in merge rules equal to the thoroughly measured Medscan false positive rate [3].

Abbreviations: TF – transcription factors; NR – nuclear receptors. The row order represents the order in which the rules were applied during the automatic curation. Rules named after the control type correspond to the merging of relation of the same type connecting two entities in the same direction but having different effect or mechanism. These rules are described in the Materials and Methods section. The percentage of relations in ResNet curated by a rule was calculated as the fraction of all relations of the type curated by a rule. The combination of all rules deletes or converts about 30% of all relations in the ResNet database.

Rule accuracy is measured as the percentage of correctly deleted relations. The accuracy was measured by the manual inspection of facts deleted by the automatic curation. About 100 deleted facts were examined for every rule. The accuracy of the cleaning rules depends entirely on the accuracy of protein classification in ResNet. Though the accuracy of many merging rules were found to be 100%, it is possible in theory that some of them can delete true positives by merging into false positive relations. Because all merging rules have a safeguard threshold of 10%, the reference count for the target relation for our random test samples did not contain such examples. Additionally, we did not perform the focused search for such cases.

**Cleaning rules**

| **Relation type** | **Mechanism** | **Protein class** | **Description** | **Percentage of relations in ResNet curated by the rule** | **Rule accuracy** |
| --- | --- | --- | --- | --- | --- |
| *PromoterBinding* |  | If regulator is not TF and not NR | delete if number of references < 5, otherwise convert to *Regulation* | 0.002% | 100% |
| *ProtModification* | Phosphorylation | If regulator is not Kinase | convert to *Regulation* with mechanism: Phosphorylation | 0.37% | 100% |
| *ProtModification* | Dephosphorylation | If regulator is not Phosphatase | convert to *Regulation* with mechanism: Dephosphorylation | 0.05% | 100% |
| *ProtModification* | Phosphorylation | If target is Ligand or Secreted Proteins | delete if number of references < 5, otherwise convert to *Regulation* | 0.04% | 99% |
| *ProtModification* | Dephosphorylation | If target is Ligand or Secreted Proteins | delete if number of references < 5, otherwise convert to *Regulation* | 0.0016% |  |
| *ProtModification* | Acetylation | If regulator is not Acetylase | convert to *Regulation* with mechanism Acetylation | 0.15% | 100% |
| *ProtModification* | Deacetylation | If regulator is not Deacetylase | convert to *Regulation* with mechanism Deacetylation | 0.013% | 100% |
| *ProtModification* | Deglycosylation | If regulator is not Deglycosylase | convert to *Regulation* with mechanism Deglycosylation | 0 | N/A |
| *ProtModification* | Glycosylation | If regulator is not Glycosyltransferase | convert to *Regulation* with mechanism Glycosylation | 0 | N/A |
| *ProtModification* | SUMO ligation | If regulator is not Ubiquitin ligase | convert to *Regulation* with mechanism Sumoylation | 0.003% | 100% |
| *ProtModification* | Geranylation | If regulator is not Geranyltranstransferase and not Farnesyltranstransferase | convert to *Regulation* with mechanism Geranylation | 0 | N/A |
| *ProtModification* | Farnesylation | If regulator is not Geranyltranstransferase and not Farnesyltranstransferase | convert to *Regulation* with mechanism Farnesylation | 0 | N/A |
| *ProtModification* | Methylation | If regulator is not Methyltransferase | convert to *Regulation* with mechanism Methylation | 0.06% | 100% |
| *ProtModification* | Ribosylation | If regulator is not ADP-rybosyl transferase | convert to *Regulation* with mechanism Ribosylation | 0.0015% | 100% |
| *Binding* |  | between two Ligands | convert to new relation type "PathwayInteraction" if the number of references > 5 | 0.59% | 90% |
| *Binding* |  | between Ligand and non-Receptor and non-GPCR and non-NR and non-Extracellular matrix and non-Secreted Protein | delete if number of references < 5, otherwise convert to *Regulation* | 0.91% | 96% |
| *ProtModification* |  | If regulator is TF or NR | delete if number of references < 5, otherwise convert to *Regulation* | 0.0015% | 99% |
| *Expression* | Degradation | If regulator is not Protease | convert to *Regulation* with mechanism Degradation | 0 | N/A |

**Merging rules**

| **Relation type** | **Relation type** | **Result relation and description** | **Percent of relations curated by the rule** | **Rule accuracy** |
| --- | --- | --- | --- | --- |
| *Expression* | *Expression* | *Expression,* relation with different effect and mechanism are merged onto relation with biggest number of references | 1.57% | 100% |
| ***MolSynthesis*** | ***MolSynthesis*** | ***MolSynthesis,*** relation with different effect and mechanism are merged onto relation with biggest number of references | 11.17% | 100% |
| *MolTransport* | *MolTransport* | *MolTransport,* relation with different effect and mechanism are merged onto relation with biggest number of references | 15.06% | 100% |
| *PromoterBinding* | *PromoterBinding* | *PromoterBinding,* relation with different effect and mechanism are merged onto relation with biggest number of references | 4.41% | 100% |
| *ProtModification* | *ProtModification* | *ProtModification,* relation with different effect and mechanism are merged onto relation with biggest number of references | 0 | N/A |
| *Regulation* | *Regulation* | *Regulation,* relation with different effect and mechanism are merged onto relation with biggest number of references | 26.54% | 100% |
| *Binding* | *ProtModification* | *ProtModification* , only if *ProtModification*  reference count is more than 10% of *Binding* count | 4.62% | 100% |
| *Binding* | *Regulation* | *DirectRegulation* with effect from *Regulation*, only if *Binding* reference count is more than 10% of *Regulation* count | 19.40% | 100% |
| *ProtModification* | *Regulation* | *DirectRegulation* with effect from *Regulation* and mechanism *ProtModification* , only if *ProtModification*  reference count is more than 10% of *Regulation* count | 8.97% | 100% |
| *Expression* | *PromoterBinding* | *PromoterBinding* with effect from *Expression* | 2.6% | 100% |
| *Regulation* | *Expression* | *Expression* with effect from the relation with bigger reference count, only if *Expression* reference count is more than 10% of *Regulation* count | 3.16% | 100% |
| *Regulation* | *PromoterBinding* | *PromoterBinding* with effect from *Regulation* | 0.23% | 100% |
| *Regulation* | *MolTransport* | *MolTransport* with effect from the relation with bigger reference count, only if *MolTransport* reference count is more than 10% of *Regulation* count | 1% | 100% |
| *Regulation* | *MolSynthesis* | *MolSynthesis* with effect from the relation with bigger reference count, only if *MolSynthesis* reference count is more than 10% of *Regulation* count | 0.31% | 100% |
| *ProtModification* | *DirectRegulation* | *DirectRegulation* with effect from *DirectRegulation* + mechanism from *ProtModification* | 0.99% | 100% |
| *Binding* | *DirectRegulation* | *DirectRegulation* with effect from *DirectRegulation* | 0.023% | 100% |

**Additional cleaning rules**

| False positive elimination | All | Deletes relations if there is relation of the same type connecting same two nodes in the opposite direction that have10 times more reference count. Only for nodes with connectivity > 50. | 0.1% | 90% |
| --- | --- | --- | --- | --- |
| Coherent loops conversion | *Expression* | Converts Expression relation to Regulation if there is a coherent loop connecting the same two nodes that contain TF binding the promoter of the target node | 5.8% | 100% |
