# Supplementary material for: Automatic pathway building in biological association networks
Source: BMC Bioinformatics. 2006 Mar 24;7:171. doi: 10.1186/1471-2105-7-171 (PMC1435941; doi:10.1186/1471-2105-7-171)
Supplement: Additional file 2 — Protein classification in ResNet and its correspondence to GO annotation. [file 1471-2105-7-171-S2.doc]

**Additional file 2.** Protein classification in ResNet and its correspondence to Gene Ontology annotation.

| ResNet class | GO classes | Protein Count |
| --- | --- | --- |
| Acetylases | acetyltransferase activity | 36 |
| ADP-ribosyltransferases | protein amino acid ADP-ribosylation | 9 |
| Cytoskeleton | structural constituent of cytoskeleton | 271 |
| Deacetylases | deacetylase activity | 6 |
| Deglycosylases | hydrolase activity, acting on glycosyl bonds | 93 |
| Extracellular matrix (EM) | extracellular matrix (sensu Metazoa) | 324 |
| Farnesyltranstransferase | farnesyltranstransferase activity | 3 |
| Geranyltranstransferases | geranyltranstransferase activity | 1 |
| Glycosyltransferases | transferase activity, transferring glycosyl groups | 197 |
| GPCR | G-protein coupled receptor activity | 2757 |
| GTP binding proteins | GTP binding | 276 |
| GTPase regulators | small GTPase regulator activity | 94 |
| Kinases | protein kinase activity | 831 |
| Ligands | cytokine activity;hormone activity;growth factor activity;opioid peptide activity;pheromone activity | 526 |
| Metabolic enzymes | oxidoreductase activity; transferase activity; | 997 |
| Methyltransferases | methyltransferase activity | 102 |
| Nuclear receptors (NR) | ligand-dependent nuclear receptor activity | 54 |
| Phosphatases | phosphoprotein phosphatase activity | 410 |
| Proteases | peptidase activity | 437 |
| Receptors | receptor activity, excluding GPCRs | 1039 |
| Secreted proteins | Extracellular, except Extracellular matrix | 239 |
| Transcription factors (TF) | transcription regulator activity; regulation of transcription, DNA-dependent | 2124 |
| Transporters | Transporter activity, except vesicle transport | 1008 |
| Ubiquitin ligases | ubiquitin protein ligase activity; ubiquitin protein ligase-like activity; | 356 |
